# Toxin genotypes, antibiotic resistance and their correlations in *Clostridioides difficile* isolated from hospitals in Xi’an, China

**DOI:** 10.1186/s12866-024-03327-z

**Published:** 2024-05-23

**Authors:** Sukai Zhang, Chen Ma, Haiyue Zhang, Congcong Zhao, Ruibing Guo, Jiahao Liu, Jing Wang, Jing Yuan, Kai Jia, Airong Wu, Yanjiong Chen, Jin’e Lei

**Affiliations:** 1 Clinical Medicine Class of 2019, Xi’an Jiaotong University, Xi’an, China; 2 Department of Clinical Laboratory, The First Affiliated Hospital of Xi’an Jiaotong University, Xi’an, China; 3 Xi’an Children’s Hospital, Xi’an, China; 4 Xi’an Gaoxin Hospital, Xi’an, China; 5 Department of Immunology and Pathogenic Biology, College of Basic Medicine, Xi’an Jiaotong University Health Science Center, Xi’an, China

**Keywords:** *Clostridioides difficile*, Toxin genes, Toxin gene expression, Antibiotic resistance

## Abstract

**Background:**

*Clostridioides difficile* is the main pathogen of antimicrobial-associated diarrhoea and health care facility-associated infectious diarrhoea. This study aimed to investigate the prevalence, toxin genotypes, and antibiotic resistance of *C. difficile* among hospitalized patients in Xi’an, China.

**Results:**

We isolated and cultured 156 strains of *C. difficile*, representing 12.67% of the 1231 inpatient stool samples collected. Among the isolates, *tcdA + B +* strains were predominant, accounting for 78.2% (122/156), followed by 27 *tcdA-B +* strains (27/156, 17.3%) and 6 binary toxin gene-positive strains. The positive rates of three regulatory genes, *tcdC*, *tcdR*, and *tcdE*, were 89.1% (139/156), 96.8% (151/156), and 100%, respectively. All isolates were sensitive to metronidazole, and the resistance rates to clindamycin and cephalosporins were also high. Six strains were found to be resistant to vancomycin.

**Conclusion:**

Currently, the prevalence rate of *C. difficile* infection (CDI) in Xi’an is 12.67% (156/1231), with the major toxin genotype of the isolates being *tcdA + tcdB + cdtA-/B-*. Metronidazole and vancomycin were still effective drugs for the treatment of CDI, but we should pay attention to antibiotic management and epidemiological surveillance of CDI.

## Background

*Clostridioides difficile*(*C. difficile*), previously known as *Clostridium difficile*, is a Gram-positive, anaerobic, spore-forming bacillus that colonizes the gastrointestinal tract of humans and animals. It is recognized as the leading cause of antimicrobial-associated diarrhoea and health care facility-associated infectious diarrhoea [1]. The spectrum of *C. difficile* infection (CDI) ranges from asymptomatic colonization and diarrhoea of varying severities to pseudomembranous colitis, toxic megacolon, and even death [2].

CDI is primarily mediated by toxins A (enterotoxin) and B (cytotoxin), which are encoded by the *tcdA* and *tcdB* genes, located in the pathogenicity locus (PaLoc) of the bacterial chromosome [3]. PaLoc also contains two genes encoding regulatory factors (*tcdR* and *tcdC*) and a holin-like gene (*tcdE*) that is involved in toxin release, although the function of *tcdC* remains controversial [4, 5]. Some *C. difficile* strains can also produce a binary toxin (CDT) encoded by the *cdtA* and *cdtB* genes, which can enhance the toxicity of TcdA and TcdB. Studies have reported that approximately 1.6-10% of *C. difficile* isolates carry binary toxin genes [6, 7]. The antibiotic resistance of *C. difficile* varies among countries and regions, but it remains sensitive to metronidazole and vancomycin [8]. Therefore, vancomycin and metronidazole are still recommended drugs for the treatment of CDI [9].

Due to the widespread and irresponsible use of broad-spectrum antibacterial drugs, the incidence of CDI has increased significantly. In the last decade, research on *C. difficile* in China has also been on the rise. Multiple-drug-resistant strains of *C. difficile* have been detected clinically, and hypervirulent strains have been identified in Beijing and other areas [10]. Wu Yuan et al. [11] showed that common clinical isolates and drug-resistance characteristics of *C. difficile* in China were generally consistent with international reports. However, the distribution of the genotypes, antibiotic resistance, and toxin genes varies in space and time.

There have been many epidemiological studies on *C. difficile* in eastern China, while research on the topic is relatively scarce in northwest China. Xi ‘an, as the economic and medical hub of northwest China, is regionally representative. Therefore, this study was conducted to investigate the current prevalence of CDI, toxin genotypes, and antibiotic resistance among inpatients in the region, by analyzing the status of patients in three tertiary hospitals in Xi’an.

## Result

### *C. Difficile* isolation and characteristics of the patients

A total of 1231 stool samples were collected in this study, and 156 strains of *C. difficile* were isolated and cultured, for a positive rate of 12.67%. There was a significant difference in the positive rate of *C. difficile* culture between the three hospitals, while there was no significant difference between the sexes. Table 1 shows that the highest positive rate of *C. difficile* culture was observed in the age group 61–70 years old (17.31%, 27/156), followed by 3–10 years old (14.55%, 8/55), and the lowest was 20–30 years old (2/44, 4.54%), but the differences were not statistically significant.

**Table 1:** Differences in positive *C. difficile* culture rates among 1231 inpatients by hospital, gender and age

Patient characteristics	*C. difficile* culture		Positive rate (%)	χ^2^	*P* value
	-	+			
**Hospital**				6.051	0.049*
A	408	70	14.64		
B	165	15	7.67		
C	477	71	12.72		
**Gender**				2.992	0.084
Female male male	550	80	11.27		
Male	379	76	14.59		
**Age (year)**				6.707	0.569
0–3	454	64	12.36		
4–10	47	8	14.55		
11–20	13	2	13.33		
21–30	42	2	4.54		
31–40	71	8	10.13		
41–50	83	13	13.68		
51–60	115	16	12.21		
61–70	129	27	17.31		
≥ 71	121	16	11.68		

Hospital A: The First Affiliated Hospital of Xi’an Jiaotong University, B: Xi’an High-tech Hospital, C: Xi’an Children’s Hospital

*Significant difference

A total of 156 patients with positive *C. difficile* cultures were hospitalized for 10.3 ± 11.8 days and were aged 31.7 ± 30.1 years. The most common departments for these hospitalized patients was the infectious diseases department (29.5%, 46/156), followed by digestive medicine (25%, 39/156), and the ICU (13.5%, 21/156). Other departments had only a few patients.

### Detection of toxin genes and toxins A and B

**Fig. 1:**
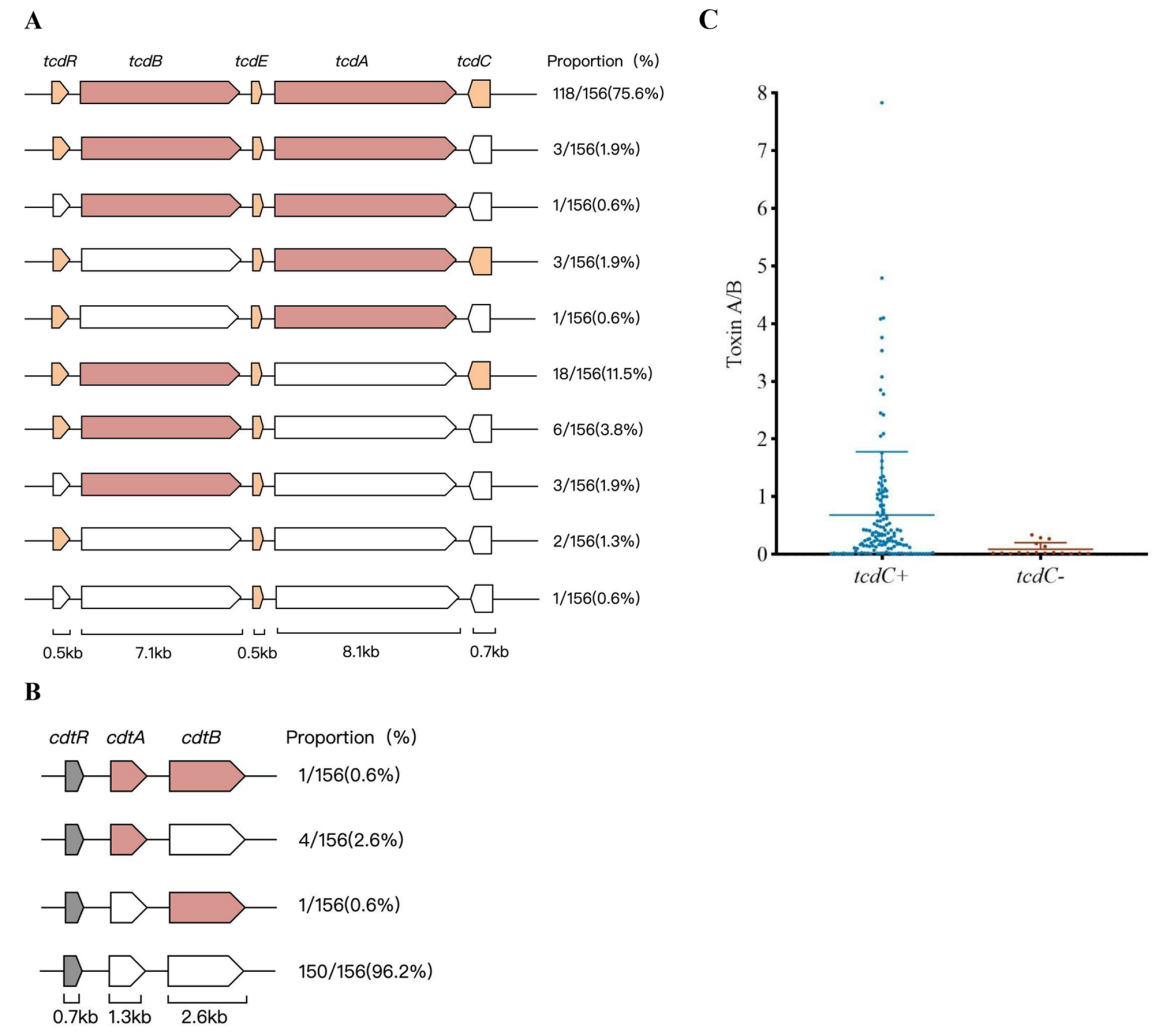
Detections of toxin genes and toxin A/B. **(A)** The PaLoc encodes the toxin A and toxin B, and three accessory proteins. The colored ones indicate that the detected isolates are positive for this gene, the colorless ones indicate a negative result, and the stripe indicates undetected. Pink is the main toxin gene, and the light orange and grey represent the accessory gene. **(B)** The CdtLoc encodes the two binary toxin genes and one accessory gene. The representation of color is the same as A. **(C)** Differences in toxin A/B secretion of different *tcdC* genes, with error bars indicating the standard deviation from the mean

The toxin gene was detected in *C. difficile* isolates, of which 98.08% (153/156) were toxigenic strains, and 1.92% (3/156) were non-toxic strains. Toxin genotyping of the strains for *tcdA* and *tcdB* showed that the toxin profiles of *tcd A + B+*, *tcd A + B-*, and *tcd A-B +* accounted for 78.2% (122/156), 2.6% (4/156), and 17.3% (27/156) of the strains, respectively. A total of 3.85% (6/156) of the *C. difficile* strains were binary toxin positive, and only one sample presented the two genes that encode the binary toxin. For all isolates that were binary toxin positive, the toxin gene profiles of *tcdA* and *tcdB* were *tcdA + B+*. As for the accessory genes, the positive rates for *tcdC*, *tcdR*, and *tcdE* in the isolates were 89.1% (139/156), 96.8% (151/156), and 100%, respectively. The distribution of toxin genes is shown in Fig. 1.

Among the 156 *C. difficile* isolates, 61 (39.1%, 61/156) were hand a positive toxin A/B test, and 95 (60.9%, 95/156) were negative/suspicious for toxin A/B. Further comparing the difference in toxin A/B secretion between *tcdC +* and *tcdC-* strains, the results showed that the A/B toxin value secreted by *tcdC +* strains (0.68 ± 1.10) was higher than that of *tcdC-* strains (0.09 ± 0.11) (*P* < 0.001), as shown in Fig. 1.

### Antimicrobial susceptibility of isolates

**Fig. 2:**
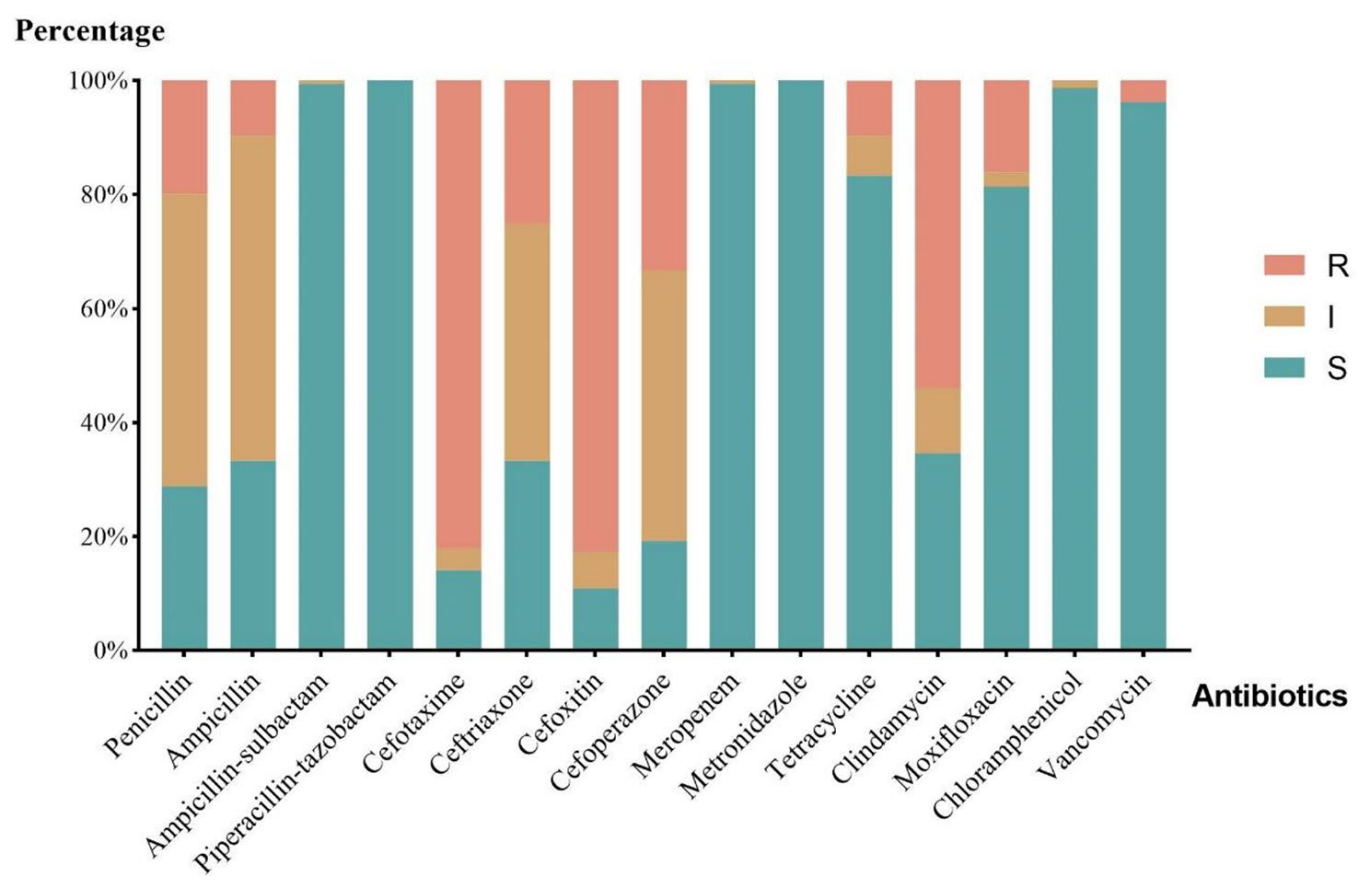
The resistance characteristics of isolated *C. difficile* strains. The name of 15 tested antibiotics is in the horizontal axis. For the vertical line, the percentages refer to the proportion of strains involved in R (Resistant), I (intermediate), and S (susceptible)

Figure 2 shows the results of the antibiotic susceptibility test. All isolates were susceptible to metronidazole and piperacillin-tazobactam and were not resistant to ampicillin/sulbactam, meropenem, or chloramphenicol. Six (3.8%, 6/156) strains were resistant to vancomycin. The antibiotic resistance rates of *C. difficile* to ampicillin (9.6%, 15/156), tetracycline (9.6%, 15/156), moxifloxacin (16.0%, 25/156), penicillin (19.9%, 31/156), and ceftriaxone (25.0%, 39/156) were low. Cefoxitin (82.7%, 129/156) was the most resisted antibiotic in the study, followed by cefotaxime (82.1%, 128/156), clindamycin (53.8%, 84/156), and cefoperazone (33.3%, 52/156).

### Differences in antimicrobial resistance according to toxin genotyping and the level of toxins produced

Table 2 shows the results of the antibiotic susceptibility testing and correlation analysis. The results showed that the resistance of strains varieds between hospitals. For cefotaxime, cefoxitin, tetracycline, and clindamycin the strains isolated from Xi’an Children’s Hospital had higher resistance rates than those from The First Affiliated Hospital of Xi’an Jiaotong University. There were differences in resistance between strains with different levels of toxin production. For ceftriaxone, cefoperazone, and tetracycline, the strains that tested positive for A/B toxin had significantly lower resistance rates than those that tested negative/suspicious. In addition, between different toxin genotypes, there were significant differences in the resistance rates to tetracycline, with *tcdA-B +* strains having higher resistance rates than *tcdA + B +* strains.

**Table 2:** Antibiotic resistance rates of *C. difficile* according to hospital, toxin production and toxin genotyping

Antimicrobial agent (number of resistant strains)	Hospital, *n* (%)				Detection of Toxin A/B, *n* (%)			Toxin genotyping, *n* (%)		
	A (*n* = 70)	B (*n* = 15)	C (*n* = 71)	*P* value	Negative/ Suspicious (*n* = 95)	Positive (*n* = 61)	*P* value	tcdA + B+ (*n* = 122)	tcdA-B+ (*n* = 27)	*P* value
Penicillin	12(17.1)	3(20.0)	16(22.5)	0.735	23(24.2)	8(13.1)	0.090	22(18)	9(33.3)	0.076
Ampicillin	9(12.9)	2(13.3)	4(5.6)	0.327	9(9.5)	6(9.8)	0.940	11(9.0)	4(14.8)	0.476
Cefotaxime	46(65.7) ^a^	13(86.7) ^a, b^	69(97.2) ^b^	<0.001*	78(82.1)	50(82.0)	0.983	101(82.8)	24(88.9)	0.570
Ceftriaxone	11(15.7) ^a^	7(46.7) ^b^	21(29.6) ^a, b^	0.021*	30(31.6)	9(14.8)	0.018*	32(26.2)	6(22.2)	0.666
Cefoxitin	48(68.6) ^a^	14(93.3) ^a, b^	67(94.4) ^b^	<0.001*	80(84.2)	49(80.3)	0.532	102(83.6)	23(85.2)	1.000
Cefoperazone	15(21.4) ^a^	9(60.0) ^b^	28(39.4) ^a, b^	0.005*	39(41.4)	13(21.3)	0.011*	38(31.1)	11(40.7)	0.337
Tetracycline	2(2.9) ^a^	2(13.3) ^a, b^	11(15.5) ^b^	0.031*	14(14.7)	1(1.6)	0.007*	9(7.4)	6(22.2)	0.032*
Clindamycin	30(42.9) ^a^	9(60.0) ^a, b^	45(63.4) ^b^	0.044*	55(57.9)	29(47.5)	0.206	63(51.6)	18(66.7)	0.156
Moxifloxacin	20(28.6) ^a^	2(13.3) ^a, b^	3(4.2) ^b^	0.001*	13(13.7)	12(19.7)	0.320	19(15.6)	5(18.5)	0.773
Vancomycin	5(7.1)	0	1(1.4)	0.187	5(5.3)	1(1.6)	0.405	3(2.5)	1(3.7)	0.555

*Significant difference

Hospital A: The First Affiliated Hospital of Xi’an Jiaotong University, B: Xi’an High-tech Hospital, C: Xi’an Children’s Hospital

a, b: If identical letters exist, they are not significant difference

### Correlation between clinical manifestations and patient characteristics

We divided the CDI patients into asymptomatic and overt infections based on clinical symptoms and analysed the correlation between them and patients’ clinical characteristics, as shown in Table 3. The results showed that patients aged 0–10 years presented with symptoms more frequently than those aged 11–50 years or > 50 years, and more frequently than the total population, all statistically significant differences. There was no statistically significant difference in the frequency of symptoms between patients aged 11–50 and patients aged > 50 years. Patients who had used proton pump inhibitors (PPIs) were significantly less likely to have symptoms than patients who had not used PPIs.

**Table 3:** Differences in clinical symptoms by age, gender, PPI and antibiotic use in *C. difficile* infection

Patient characteristics		Clinical symptoms			Positive rate (%)		χ^2^	*P* value
		-	+					
**Total**	51			105		67.3		
**Age(year)**							36.57	<0.001^*^
0-10^a^		6	66		91.7		15.59^c^	<0.001^*, c^
11-50^b^		12	13		52.0		2.23^c^	0.175^c^
>50^b^		33	26		44.1		9.71^c^	0.002^*, c^
**Gender**							0.95	0.331
Female		22	54		71.1			
Male		29	51		63.8			
**PPI**							5.15	0.023^*^
Yes		20	23		53.5		2.81^c^	0.107^c^
No		31	82		72.6		0.86^c^	0.421^c^
**Antibiotics**							0.52	0.469
Yes		36	68		65.4			
No		15	37		71.2			

*Significant difference

a, b: If identical letters exist, they are not significant difference

c: Compared with total (156)

PPI: Proton pump inhibitors

## Discussion

CDI is a significant public health concern worldwide. In China, research about *C. difficile* has grown significantly in the last decade, and a meta-analysis of the literature showed that up to 14% of patients with diarrhoea tested positive for *C. difficile* [12]. In our study, we cultured 156 strains of *C. difficile* (12.67%) from faecal specimens of 1231 hospitalized patients. The positivity rate varied significantly across the three participating hospitals. We did not find a significant difference in the positivity rate between sexes or age groups. The incidence rate of CDI in our study was lower than the reported overseas (20-32%) [13, 14], and similar to the incidence rate in eastern regions of China, such as Zhejiang, Shanghai, and Shandong (10-14%) [15–17]. Differences in CDI may be related to geographical distribution, infection control policies, and antibiotic use regulations [18]. Although the incidence rate of CDI was higher among elderly patients in our study, the difference in incidence rate between other age groups was not statistically significant. This result contradicts the traditional belief that older age is a risk factor for CDI, which may be due to the widespread use of broad-spectrum antibiotics in China, leading to the continuous rejuvenation of CDI risk factors [15, 16, 19].

*C. difficile* produces TcdA, TcdB, and binary toxins (CDT), which are encoded by the *tcdA*, *tcdB*, and *cdtA/B* genes, respectively [20]. In this study, the toxin-gene-carrying rate of the isolated *C. difficile* strains was 98.08% (153/156), which is higher than the 82-90% isolation rate of poison-producing strains found in eastern China [15–17], and higher than the 63-94% isolation rate found in South Korea, Japan and other East Asian countries [17–19]. The main toxin genotype was found to be *tcdA + tcdB + cdtA-/B-*, accounting for 74.3% (116/156), which is consistent with the 69-77% ratio in eastern China [15–17]. This ratio is lower than the proportion of 77-94% in East Asian countries such as South Korea [21–23], and higher than the proportion of document coverage in Greece, Iran, Saudi Arabia, and Thailand (27-65%) [18, 24–26], indicating that the distribution of toxin genes varies greatly in between countries and regions. In this study, we isolated 27 tcdA-B + strains (17.3%, 27/156), and according to relevant literature, they may have higher resistance rates to a variety of antibiotics [27]. At the same time, six strains carrying the binary toxin genes were detected in this study (3.85%, 6/156), while no binary toxin genes were detected in studies conducted in Shanghai in 2007–2008, Zhejiang in 2014–2016 or Beijing in 2018 [15, 16, 28]. The detection rate in Asian countries such as South Korea and Thailand is also relatively low [21, 26], whereas the detection rates have been higher in Europe, such as 51% in Greece and 34% in the Czech Republic [22, 29]. The expression of binary toxin genes is believed to be associated with higher toxicity of TcdA and TcdB, higher spore production rates, and more severe diseases [30, 31]. One sample of a *cdtA + B +* strain from an elderly woman who was admitted to the infection unit for 25 days with a diagnosis of cirrhosis of the liver with ascites, was isolated in this study. In recent years, strains carrying CDT have been detected in China [32, 33], showing the need for attention and monitoring. In this study, the regulatory genes *tcdC*, *tcdR*, and *tcdE* on PaLoc were detected at rates of 89.1% (139/156), 96.8% (151/156), and 100%, respectively. Although the function of TcdR as a positive regulator has been determined, the roles of TcdC and TcdE are still under investigation [5]. We found that the secretion of toxin A/B in *tcdC* positive strains was significantly higher than that in *tcdC* negative strains, indicating that *tcdC* may play a positive regulatory role in the secretion of toxin A/B. The results of B Dupuy [34] and Kate E Dingle [35] showed that *tcdC* negatively regulates the expression of *tcdA* and *tcdB* genes, while the results of Prerna Vohra [36] and Michelle Merrigan [37] showed that *tcdC* gene regulates toxin secretion but is not strictly inhibitory. In addition, the study by Cartman ST [38] did not observe a relationship between toxin gene expression and *tcdC* genotypes. Therefore, further research on the function of the *tcdC* gene is needed.

According to the updated 2017 guidelines in the United States [9], vancomycin is the first-line clinical treatment recommended for CDI, while metronidazole is recommended for use when vancomycin cannot be obtained. The results of drug susceptibility testing in this study show that all isolates were sensitive to metronidazole, consistent with some domestic and foreign reports [16, 21, 25, 26, 35, 36]. For vancomycin, we detected six resistant strains (3.8%, 6/156), with five strains coming from patients aged 60–70 years old, who had underlying diseases such as rectal cancer, renal cancer, lymphoma, and intestinal tuberculosis. Since the Clinical and Laboratory Standards Institute (CLSI) standards in the United States have not given a recommended resistance breakpoint for vancomycin, this study used the resistance breakpoint proposed by the European Committee on Antimicrobial Susceptibility Testing (EUCAST), which defines the critical value of drug sensitivity minimum inhibitory concentration (MIC). Strains resistant to vancomycin and metronidazole have also been detected at home and abroad. The longitudinal monitoring of antibiotic resistance in 22 European countries conducted by J. Freeman et al. [39] showed that 0.87% (8/918) of strains were resistant to vancomycin and 0.11% (1/916) were resistant to metronidazole. T. Dilnessa et al. [40] included 15 cross-sectional studies from Iran, China, the United States, Poland, and other countries in a meta-analysis of antibiotic resistance rates, which showed that the resistance rates for vancomycin and metronidazole were 3% (83/2755) and 5% (138/2753), respectively. Although both antibiotics can still be used for the treatment of CDI in the region, continuous monitoring of drug resistance and strict management of antibiotic use are needed to reduce the emergence of drug-resistant strains. We found that all isolates were not resistant to piperacillin/tazobactam, ampicillin/sulbactam, and meropenem. Due to these drugs’ distribution in the intestine and their impact on the normal intestinal flora, they may increase the risk of CDI and even worsen the condition [16]., so, these antibiotics are not routinely recommended for the treatment of CDI. Meanwhile, the resistance rates to traditional antibiotics such as chloramphenicol, tetracycline, ampicillin, and penicillin were relatively low, possibly due to the decreasing use of these traditional antibiotics in tertiary hospitals in recent years in China. In this study, the isolates had high resistance rates to third-generation cephalosporin antibiotics and clindamycin, which are high-risk drugs for CDI [40, 41]. This suggests that, in the region, the use of these drugs may be a risk factor for CDI and that the use of clindamycin and cephalosporins should be limited [9]. The relative risk associated with the use of specific antibiotics and their correlation with CDI depends on the prevalence of highly resistant strains to specific antibiotics in the local area [41]. Although fluoroquinolones are also considered high-risk drugs for CDI [42], the resistance rate of the isolates to moxifloxacin in this study was 16.0% (25/156), which is lower than the reported resistance rates of 25-93% in other domestic and foreign reports [32, 39, 43]. As a result, the use of moxifloxacin may not be the main risk factor for CDI in the region though further research is needed to verify this.

By comparing their rates of resistance to different antibiotics, we showed that for some antibiotics, the resistance rate of strains isolated from Xi’an Children’s Hospital was higher than that of strains from The First Affiliated Hospital of Xi’an Jiaotong University. This may be due to the different antibiotic use habits and the antibiotics used for different age groups in the two hospitals. We also found that *tcdA-B +* strains showed significantly higher resistance to tetracycline than *tcdA + B +* strains, suggesting that *tcdA-B +* strains have higher resistance to some antibiotics. These findings are consistent with previous reports [15, 25, 26], indicating that toxin gene typing may be valuable in handling antibiotic use in CDI patients. In addition, the isolates that tested positive for A/B toxin were resistant to ceftriaxone, cefoperazone, and tetracycline significantly less often than the negative/suspicious isolates, suggesting that stronger strains may be less resistant to some antibiotics. The results from a study by T. Saber [25] showed that toxigenic strains had higher resistance to most antibiotics than non-toxigenic strains, indicating that further institutional research is needed to clarify the relationship between *C. difficile* toxin production and drug resistance phenotypes.

Our correlation analysis of the clinical characteristics and infectious symptoms of patients with CDI showed that clinical symptoms most often appeared in patients aged 0–10 years. However, it has been traditionally believed that CDI in infants and young children is mostly non-pathogenic due to the protection provided by breastfeeding and the lack of toxin receptors [44]. Previous studies have reported a high prevalence of coinfection with *C. difficile* and other gastrointestinal pathogens in children with diarrhoea (23.2 − 67%) [45]. As we did not test for other pathogens in our study, it is possible that coinfection with other pathogens contributed to the development of clinical symptoms in our CDI patients. Our findings also revealed that the use of PPIs was associated with a lower probability of symptoms compared to the non-use of PPIs. Although previous studies have shown that the use of PPIs significantly increases the risk of CDI, most of them have been observational studies that could not establish a causal relationship [46]. Moreover, the 2017 updated guidelines in the USA stated that there was not enough evidence to suggest that stopping PPI use is an effective measure to prevent CDI [9]. Therefore, the relationship between PPIs and the clinical symptoms of CDI patients is unclear.

This study enhances our understanding of the prevalence of CDI in the Xi’an region, but it had some limitations. First, the *C. difficile* isolates used in this study were collected over a period of time, and the epidemiology of CDI is dynamic. Therefore, the distribution of toxin genotypes and antibiotic resistance may have slightly biased our data analysis. Second, fidaxomicin, as one of the preferred antibacterial drugs for the treatment of CDI, was not evaluated due to the unavailability of the test substance. Finally, since there was no continuous monitoring of patient disease progress, the clinical data collected only reflected the symptoms of patients at a certain point in time, and there may be biases in the correlation analysis of clinical symptoms. Future investigations should be designed to avoid these shortcomings.

## Conclusions

This study contributes to the understanding of the prevalence of CDI, toxin genotyping, and antibiotic resistance in clinical isolates of *C. difficile* in Xi’an, China. In summary, the CDI incidence rate in the study area was 12.67% (156/1231), with *tcdA + tcdB + cdtA-/B-* as the main toxin genotype. A total of 27 *tcdA-B +* strains and 6 strains positive for binary toxin genes were isolated. Vancomycin and metronidazole were still effective in treating CDI in the region, but antibiotic management, especially with clindamycin and cephalosporins, should be given full attention to reduce the incidence of CDI and prevent the emergence of new resistant strains. More, more in-depth studies are recommended to explore the function of the tcdC gene, clarify the relationship between toxin production and the resistance phenotype of *C. difficile*, evaluate the relationship between PPI use and CDI clinical symptoms, and monitor the rapidly changing CDI epidemiology.

## Methods

### Study design and sample collection

This cross-sectional study was carried out in the First Affiliated Hospital of Xi’an Jiaotong University, Xi’an High-tech Hospital, and Xi’an Children’s Hospital from July to October 2020. We collected 2–3 ml of fresh unformed stool samples from hospitalized patients with suspected antibiotic-associated diarrhoea and stored them at − 20 °C. Isolation, culture, and identification of *C. difficile* were performed within one week. We used the electronic medical record systems of the hospitals to collect relevant clinical information about patients, including sex, age, underlying illness, clinical symptoms drug use, length of stay, etc.

### Culture and identification of *C. Difficile*

All faecal samples from patients were cultured on CHROMagar chromogenic plates for *Clostridium difficile* (B.N.: 20,210,127, Shanghai Xinzhong Bioengineering Co., Ltd), incubated in anaerobic bags, and placed in a 35 °C thermostatic incubator for 24–48 h [47]. *C. difficile* colonies show typical grey to black, irregular, and rough colonies. We selected suspicious colonies by plating on chromogenic medium and used matrix-assisted laser desorption/ionization time-of-flight mass spectrometry (MALDI-TOF MS French Bio-Merieux) for identification [48]. The identification result had to be *C. difficile* with a confidence level of 99.9% and a green box mark.

### Detection of *C. Difficile* toxin genes

DNA was extracted from the isolates using a bacterial DNA extraction kit (Omega D3350, China). Using the extracted DNA as a template, polymerase chain reaction (PCR) was performed for the identification of the *tcdA*, *tcdB*, *tcdC*, *tcdE*, *tcdR*, *cdtA*, and *cdtB* genes with, the primer sequences in Table 4 [49]. The 50µL reaction system contained 25 µL of 2×Taq Master Mix, 2.5µL of upstream primer and downstream primer, 5 µL of DNA template, and 15 µL of ddH_2_O. The PCR parameters were as follows: 5 min at 94 °C for initial denaturation; 35 cycles of 94 °C for 1 min, 57 °C for 5 min and 72 °C for 50 s; and a final extension cycle of 5 min at 72 °C. Two microlitres of amplification product was placed in a 2% agarose gel, and electrophoresed for 45 min under 150 V voltage, and the results were observed using the gel imaging system.

**Table 4:** Primes used for the detection of *C. difficile* toxin genes

Target gene	Prime	Oligonucleotide sequences (5’ – 3’)	Product length (bp)
*tcdA* ^*a*^	*tcdA-F* *tcdA-R*	AGATTCCTATATTTACATGACAATAT GTATCAGGCATAAAGTAATATACTTT	369
*tcdB* ^*a*^	*tcdB-F* *tcdB-R*	TGATGAAGATACAGCAGAAGC TGATTCTCCCTCAAAATTCTC	688
*tcdC* ^*a*^	*tcdC-F* *tcdC-R*	AAAAGGGAGATTGTATTATGTTTTC CAATAACTTGAATAACCTTACCTTCA	479
*tcdR* ^*b*^	*tcdR-F* *tcdR-R*	AAAAGCGATGCTATTATAGTCAAA CCTTATTAACAGCTTGTCTAGAT	300
*tcdE* ^*b*^	*tcdE-F* *tcdE-R*	GTTTAAGTGCAATAAAAAGTCGTA GGTAATCCACATAAGCACATATT	262
*cdtA* ^*a*^	*cdtA-F* *cdtA-R*	GGGAAGCACTATATTAAAGCAGAAGC CTGGGTTAGGATTATTTACTGGACCA	200
*cdtB* ^*a*^	*cdtB-F* *cdtB-R*	TTGACCCAAAGTTGATGTCTGATTG CGGATCTCTTGCTTCAGTCTTTATAG	260

a: The primers used in the study were designed by Primer-BLAST

b: The primers used in the study were from Reference 48

### Fluorescence immunoassay of toxins A and B

The *C. difficile* toxins A and B of *C. difficile* were isolated by enzyme-linked fluorescence immunoassay (ELFA) and VIDAS automated instrument (B.N.: 1,009,414,570, French Bio-Merieux). Solidphase tubes were coated with rabbit-derived polyclonal antitoxin A antibody and mouse-derived monoclonal antitoxin B antibody to *C. difficile*. The reagent strip contained: 0.05 mol/L TRIS buffer, conjugate (biotin-labelled mouse-derived monoclonal anti-*C. difficile* toxin A antibody and biotin-labelled mouse-derived monoclonal anti-*C. difficile* toxin B antibody), tracer (alkaline phosphatase-labelled streptavidin), and substrate (4-methylumbelliferyl phosphate). The test was performed according to the manufacturers’ specifications. Fluorescence intensities for A/B toxin of < 0.13, ≥ 0.13 to < 0.37, and ≥ 0.37 were considered negative, equivocal, and positive, respectively [47].

### Antimicrobial resistance testing

The MIC of 15 common clinical antibacterial drugs was determined by anaerobic biochemical drug sensitivity test card TDR ANA-96 (B.N.:20,211,226, Hunan Mindray Medical Technology Co., Ltd.). Antimicrobial drugs include penicillin, ampicillin, ampicillin-sulbactam, piperacillin-tazobactam, cefotaxime, ceftriaxone, cefoxitin, cefoperazone, meropenem, metronidazole, tetracycline, clindamycin, moxifloxacin, chloramphenicol, and vancomycin. The MIC breakpoints of vancomycin were based on the EUCAST recommendation [50], and the CLSI recommendation [51].

### Data analysis

The statistical software used in this study was SPSS 20.0. The measurement data are expressed in$\stackrel{-}{ x}\pm s$ (mean ± standard deviation) and were compared using the test; The enumeration data are expressed in cases or percentages, and were compared using the chi-square test or Fisher’s exact test. Bonferroni correction was done for pairwise comparisons after multiple comparisons of the chi-square test; *p* < 0.05 was considered statistically significant.

## Data Availability

The data generated and/or analyzed during the current study are available from the corresponding author on reasonable request.

## Abbreviations

*C. difficile*: *Clostridioides difficile*
CDI: *C. difficile* infection
PaLoc: pathogenicity locus
PPI: proton pump inhibitor
PCR: polymerase chain reaction
ELFA: enzyme-linked fluorescence immunoassay
MIC: minimum inhibitory concentration
EUCAST: European Committee on Antimicrobial Susceptibility Testing
CLSI: Clinical and Laboratory Standards Institute
